# Clinical and molecular characterization of chondrodysplasias in a cohort of Egyptian patients

**DOI:** 10.1038/s41598-025-22794-6

**Published:** 2025-10-29

**Authors:** Miral M. Refeat, Rasha M. Elhossini, Heba Amin Hassan, Mona S. Aglan, Alice Abdelaleem, Mona L. Essawi

**Affiliations:** 1 Department of Medical Molecular Genetics, Human Genetics and Genome Research Institute, National Research Centre, Cairo, Egypt; 2 Department of Clinical Genetics, Human Genetics and Genome Research Institute, National Research Centre, Cairo, Egypt; 3 Center of Excellence of Human Genetics (CEHG), National Research Centre, Cairo, Egypt; 4 Medical Molecular Genetics Department, Human Genetics and Genome Research Institute, National Research Centre, Cairo, 12311 Egypt

**Keywords:** Skeletal dysplasias, Diastrophic dysplasia, SLC26A2, Novel variants, Exome sequencing, Molecular medicine, Diseases

## Abstract

Skeletal dysplasias (SDs) are a broad and heterogeneous group of genetic disorders primarily affecting bone and collagen development. Diastrophic dysplasia (DTD) is a rare autosomal recessive chondrodysplasia caused by biallelic variants in the *SLC26A2* gene. The current study aims to assess the clinical and molecular findings in Egyptian patients with DTD. This study enrolled fifteen patients who were clinically diagnosed with DTD. Exome sequencing identified nine homozygous variants in the *SLC26A2* gene across ten patients; five of these variants were novel (p.Cys78Gly, p.Leu132Pro, p.Asp177Tyr, p.Thr546Ala, and p.Leu554Phe), while four had been previously reported. Novel variants were confirmed using Sanger sequencing and were predicted to be disease-causing based on in silico analyses and structural protein modeling. The exome sequencing analysis did not reveal any additional candidate genes that could contribute to the clinical phenotype in patients with negative *SLC26A4* causative variants. Our findings expand the spectrum of variants associated with DTD, which may aid in early diagnosis and counseling for affected families. Further studies are needed to confirm computational predictions of novel variants and their consequences in disease mechanisms, and to identify causative genes in the undiagnosed cases.

**Supplementary Information:**

The online version contains supplementary material available at 10.1038/s41598-025-22794-6.

## Introduction

Genetic skeletal disorders are heterogeneous and comprises 771 entries associated with 552 genes according to the latest 11th revision of the “Nosology of genetic skeletal disorders”^1^, reflecting the increase in molecular delineation of new disorders due to advances in DNA sequencing technology.

Skeletal dysplasias (SDs) are associated with abnormalities in the growth and maintenance of cartilage and bone structures, resulting in variable disease severities ranging from short stature to severe developmental syndromes and perinatal lethality^2^. Although, these disorders are mostly rare, the incidence of SDs is approximately 1 in every 3,000 to 1 in every 5,000 live births^1^. SDs can be sorted into three groups: chondrodysplasias, osteodysplasias and dysostosis^3^. Genetic heterogeneity and overlapping of clinical features make the diagnosis of SDs challenging^4^. Sanger sequencing is considered as a common technique for identification of variants in the different genes known to cause SDs, however, exome sequencing (ES) is a practical tool to elucidate the variants underlying such complex conditions^5^.

Chondrodysplasias are related to cartilage disorders that result in skeletal developmental abnormalities, which in turn leads to bone and joint deformities in the limbs, trunk and skull^6^. Diastrophic dysplasia (DTD), (OMIM #222600) is a rare autosomal recessive chondrodysplasia, caused by pathogenic biallelic variants in the sulfate transporter gene (*SLC26A2*) gene (OMIM #606718), located on 5q32. This gene is also known as diastrophic dysplasia sulfate transporter (DTDST) gene^7^. *SLC26A2* gene encodes a solute carrier transporter transmembrane protein of 739 amino acids, and posses’ three exons, only two of them are coding ones. SLC26A2 protein transports sulfate ions across cell membranes, which mediates sulfate uptake into chondrocytes in order to maintain adequate sulfation of proteoglycans needed for cartilage development^8^. This protein is essential for chondrocyte proliferation, differentiation and cell size expansion^9^. Pathogenic variants in *SLC26A2* gene lead to a wide spectrum of both lethal and non-lethal SDs. Pathogenic homozygous or compound heterozygous variants in the *SLC26A2* gene were reported in association with DTD^1^.

The prevalence of DTD is estimated to be 1 in 100,000 to 1 in 300,000 live births, with higher prevalence in Finnish people reaching 1 in 33,000; due to a higher carrier rate in Finland^10^.The first symptoms of DTD are observed at birth, in the form of defects in the cartilage buildup process, affecting skeletal formation, which could be associated with respiratory complications leading to increased mortality in the neonatal period^11^. The characteristic features of DTD are limb shortening, spinal deformities, large joint contractures, ulnar deviation of fingers, sandal gap between the first and second toes, bilateral clubfoot, and abduction of the thumbs (also known as “Hitchhiker’s thumbs). Additionally, hearing loss has been reported in 66% of the cases and IQ is usually normal. Follow up of DTD patients showed bone complications, such as an earlier presentation of osteoarthritis and progressive spinal deformity^12^. DTD is diagnosed according to clinical characteristics, radiological findings and molecular studies. Prenatal diagnosis can be performed by ultrasound, followed by genetic testing^13^. Currently, there is no curative treatment for DTD, patients are mainly managed with physiotherapy and corrective orthopedic surgeries^7^.This study aims at identifying the molecular causative variants of clinically suspected DTD patients referred to specialized clinics in Egypt, and highlighting the clinical manifestations.

## Subjects and methods

In this study, fifteen Egyptian patients (6 Females and 9 Males) from unrelated families were included. Their ages at presentation ranged from 4 days to 17 years. The study was approved by the Medical Research Ethics Committee, NRC. An informed consent was signed by the parents. Affected individuals were subjected to detailed clinical examination, anthropometric measurements and radiological examination. Family pedigrees were performed.

### Molecular analysis

#### Genomic DNA extraction

Genomic DNA was extracted from peripheral blood samples of all participants using a GeneJET Whole Blood Genomic DNA Purification Mini Kit (Thermo Scientific, USA) according to the manufacturer’s protocol, DNA concentration and purity was determined using Qubit™ Fluorometer (Thermo Fisher Scientific, Inc.).

## Exome sequencing (ES)

Library preparation and (ES) run for genomic DNA of affected cases was performed using the Illumina platform according to the manufacturer’s protocol. Raw sequencing reads were aligned to the human reference genome (GRCh37/hg19) using Burrows- Wheeler Aligner^14^. Duplicate read marking, local realignment, SNP/Indel, base quality score recalibration and variant calling were performed using Freebayes and Genome Analysis Tool Kit (GATK) platform. The sequencing depth and coverage for the tested sample was calculated based on the alignments^15^. Sequencing run included in-process reference sample(s) for quality control, which passed thresholds for sensitivity and specificity^16^. Variants that were identified with a frequency of > 1% in the 1,000 Genomes Project (http://browser.1000genomes.org) were excluded. Copy number variations (CNVs), defined as single exon or larger deletions or duplications (Del/Dups), were detected from the sequence analysis data using a proprietary bioinformatics pipeline, which processes aligned sequence reads^17^.

## Bioinformatics prediction of variant pathogenicity

In-silico bioinformatics tools were used to identify the pathogenicity and frequency of the variants as well as to predict the potential damaging effect of these detected variants on the stability and expression of SCA26A2 protein which will in turn affect its function. These tools included Sorting Intolerant Form Tolerant (SIFT) (http://sift.jcvi.org), polymorphism phenotyping version 2 (Polyphen V2) (http://genetics.bwh.harvard.edu/pph2), dbSNP, REVEL, MetaLR and phyloP100^18,19^. These tools rely on conservation scores across different species, and physio-chemical differences between the wildtype and mutant amino acid residues.

Detected variants were classified into pathogenic, likely pathogenic, uncertain significance, and benign according to the American College of Medical Genetics and Genomics and the Association for Molecular Pathology (ACMG/AMP) and according to the latest recommendations for PP3 and BP4 ACMG/AMP criteria using individual tools^20,21^.

## Sanger sequencing for variants’ validation

To confirm the novel variants in affected participants, DNA fragments were amplified using polymerase chain reaction (PCR), with primer that were designed according to Primer3 tool (http://bioinfo.ut.ee/primer3-/). PCR primer sequences are available upon request. Amplified fragments were sequenced using ABI 3500 Genetic Analyzer according to the manufacturers’ protocols. Chromatograms were aligned by BLAST online software.

## Results

### Clinical findings

**Fig. 1:**
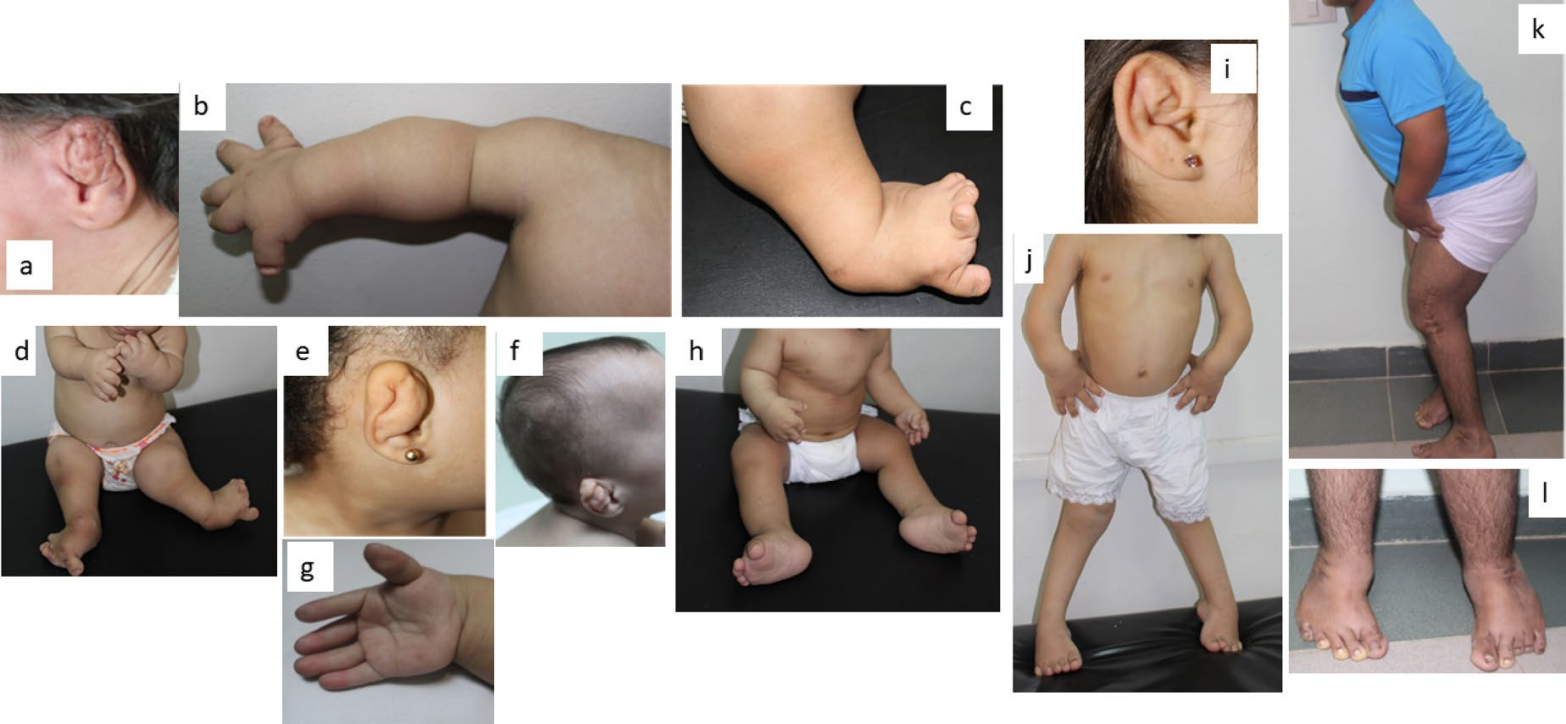
Clinical Manifestations of the studied patients. (**a**) P3 at the age of 4 days; note the cauliflower ear deformity. (**b**&**c**) Right upper limb and left leg of P2 at the age of 1 year and 4 months revealing brachydactyly, hitchhiker thumb, talipes, and broad medially displaced big toe. Note the nail dysplasia. (**d**&**e**) P4 at the age of 2 years showing rhizomelia, brachydactyly, hitchhiker thumbs, hypoplastic nails, and bilateral talipes. Note the deformed cystic ear. (**f**&**g**) P11 at 4 months of age showing deformed cauliflower ear and adducted hitchhiker thumb. (**h**) P12 at the age of 9 months showing pectus carinatum, rhizomelia, flat feet and broad medially displaced big toes. (**i**&**j**) P5 at the age of 4 years showing pectus excavatum, broad joints, bilateral genu valgum and bilateral talipes. Note the mild cystic change of ear pinnae. (**k**&**l**) P13 at the age of 17 years showing forward leaning, scar of knee operation, metatrsus adductus foot deformities, shortening of 3rd, 4th, and 5th toes in left foot and short 4th and 5th toes in right foot with bilateral broad toes and dysplastic nails.

**Fig. 2:**
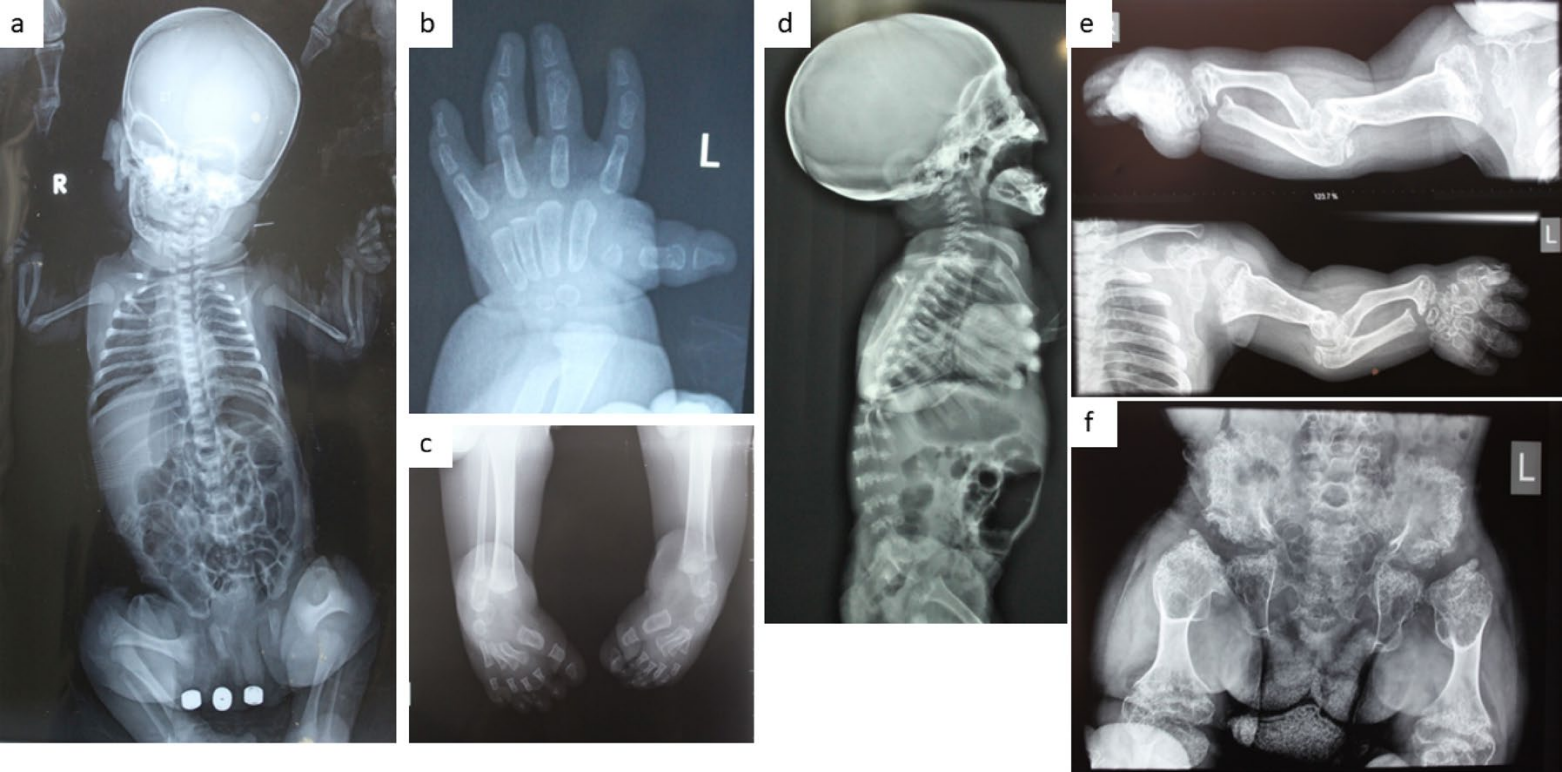
Radiological findings in the studied patients. (**a**) Whole body X ray of P1 at the age of 1 week revealing small vertebrae, flexed upper and lower limbs, dumbell shaped femora and dislocated knees. (**b**) Left hand X ray of P2 at the age of 2 years showing brachydactyly and hitchhiker thumb due to hypoplastic 1st metacarpal. (**c**) Feet x ray of P2 showing bilateral club feet. (**d**) X ray skull and spine (lateral view) of P11 at the age of 4 months showing kyphosis, small vertebrae and severe platyspondyly. (**e**) Upper limbs x ray of P4 at the age of 7 years showing short broad deformed long bones. Notice the elbow deformities. (**f**) Pelvis and hip x ray of P4 showing hypoplastic pelvis, flat irregular acetabulum, absent head of femurs, short broad femorae with metaphyseal cupping and broadening.

A total of fifteen cases (9 males and 6 females) descending from unrelated Egyptian families with a provisional clinical diagnosis of DTD were enrolled in the current study. Clinical data of the cases is shown in Table 1 and Supp Table 1. Fourteen subjects were the offspring of consanguineous parents. The studied family pedigrees were suggestive of autosomal recessive inheritance. All presented with variable degrees of skeletal deformities that were noticed at birth with a progressive course (Figs. 1 and 2). Disproportionate short stature mainly affecting the limbs was noted in all patients. Cardiac examination was normal for all patients apart from P4 who had a closing PDA and ASD. P1 has umbilical hernia. Short limbs were seen prenatally by ultrasound examination at the late trimester in two patients (P1, P8). P11 was initially a twin pregnancy, but one fetus was aborted during the pregnancy. The mother of P14 experienced a threatened abortion and received treatment.

**Table 1:** Clinical characteristics of the studied cases with homozygous variants in *SLC26A2* gene.

Criteria	P1	P2	P3	P4	P5	P6	P7	P8	P9	P10
Age at examination	7 months	1 year and 4 months	4 days	7 years	2 years	8 months	3 years	1 years and 7 months	11 months	4 days
Sex	M	F	F	F	F	F	F	M	M	M
Parental consanguinity	+ve	+ve	+ve	+ve	+ve	+ve	+ve	+ve	+ve	+ve
Similarly affected family members	3 sibs who died during the 1st year of life	-ve	-ve	-ve	A 5 years old cousin	Cousin	-ve	1 sib who died early in life	2 sibs died	-ve
Motor milestones	Delayed	Delayed	Not yet	Delayed	Delayed	Delayed	Delayed	Delayed	Delayed	Not yet
Height SDS	-4.0	-4.6	-7.6	-8.5	-5.0	-4.2	-2.5	-3.6	-4.1	-7.3
Disproportionate short stature	Short limbs dwarfism	Short limbs dwarfism	Short limbs dwarfism	Short limbs dwarfism	Short limbs dwarfism	Short limbs dwarfism	Short limbs dwarfism	Short limbs dwarfism	Short limbs dwarfism	Short limbs dwarfism
Segmental shortening of long bones	rhizomelia	rhizomelia	rhizomelia	rhizomelia	acromesomelia	rhizomelia	rhizomelia	rhizomelia	rhizomelia	rhizomelia
Glabellar hemangioma	-ve	-ve	+ve	-ve	-ve	-ve	-ve	-ve	-ve	-ve
Ear pinnae	Deformed and low set	Cystic swelling	Cauliflower shape Low set	Cauliflower shape due to ear cartilage enlargement	Cystic swelling & mildly infected ear mass	Swelling of the external ears	Cystic swelling	Cystic swelling	Cauliflower shape due to ear cartilage enlargement	Cystic swelling
Cleft palate	-ve	-ve	-ve	-ve	-ve	-ve	+ve	+ve	-ve	-ve
Adducted (Hitchhiker) thumbs	+ve	+ve	+ve	+ve	+ve	+ve	+ve	+ve	+ve	+ve
Brachydactyly	+ve	-ve	+ve	+ve	+ve	+ve	+ve	+ve	+ve	+ve
Nails	dysplastic	Normal	Normal	dysplastic	dysplastic	dysplastic	dysplastic	dysplastic	dysplastic	dysplastic
Talipes	+ve	+ve	+ve	+ve	+ve	+ve	+ve	+ve	+ve	+ve
Broad 1st toes	+ve	-ve	-ve	+ve	-ve	+ve	+ve	+ve	+ve	+ve
Joint dislocations	-ve	Elbows, hip and knees	Hip and knees	Elbows, interphalangeal joints	-ve	Elbows	Abnormal position of ankles and wrists	Elbows	Interphalangeal joints	Elbows, interphalangeal joints
Contracture deformities	Knees	Elbows and knees	Elbows	Knees	-ve	Elbows and knees	Elbows and knees	Elbows and knees	Elbows and knees	Elbows and knees
Short broad Long bones (radiological)	+ve	+ve	-ve	+ve	-ve	+ve	+ve	+ve	+ve	+ve
Epiphysis (radiological)	Small, absent head of femurs	Small	Small, absent head of femurs	Small hypoplastic, absent head of femurs	Flat	Deformed	Deformed	Deformed	Deformed	Deformed
Metaphysis (radiological	Mildlybroad	Broad	Broad	Broad	Broad	Broad	Broad	Broad	Broad	Broad
Spine	Scoliosis	Normal	Normal	scoliosis	Normal	Scoliosis	Scoliosis	Scoliosis	Scoliosis	Soliosis
Vertebrae (radiological)	Small and flat	Small and flat	Small, flat	Platyspondyly, anterior beaking	Platyspondyly	Decrease of the vertebral interpedicular distance	Irregular vertebral end plate	Irregular vertebral end plate	Irregular vertebral end plate	Irregular vertebral end plate
Flat acetabulum (radiological)	+ve	+ve	+ve	+ve	+ve	+ve	+ve	+ve	+ve	+ve
Others	-	Prominent chest	-	Prominent chest	Pectus excavatum	Abnormal skull - Pectus excavatum	Skull deformities - Pectus excavatum	Bell shaped chest	Abnormal skull with absent head of femurs - Pectus excavatum	Abnormal skull - Prominent chest

## Molecular findings

After filtration of NGS data, ES analysis of the affected patients revealed nine potential homozygous variants in *SLC26A2* gene in ten candidates (73%), five variants were novel and four were previously reported.

Novel missense variants, (c.1636 A > G/p.Thr546Ala) and(c.1660 C > T/p.Leu554Phe), located in exon3, were detected in patients 3 and 4. The variants (c.232T > G/p.Cys78Gly), (c.529G > T/p.Asp177Tyr), and (c.395T > C, p.Leu132Pro) in exon 2, were detected in patients 6, 8 and 10 respectively.

Meanwhile, the four previously reported variants were identified in exon3. Three missense variants; the (c.1157 C > T/p.Ala386Val) variant was detected in patients 1 and 2, the (c.1120 C > T/p.Pro374Ser) variant in patient P5, and the (c.835 C > T/ p.Arg279Trp) variant in patient P7. One in-frame deletion(c.1020_1022delTGT/p.Val341del) variant was detected in patient P9.

**Fig. 3:**
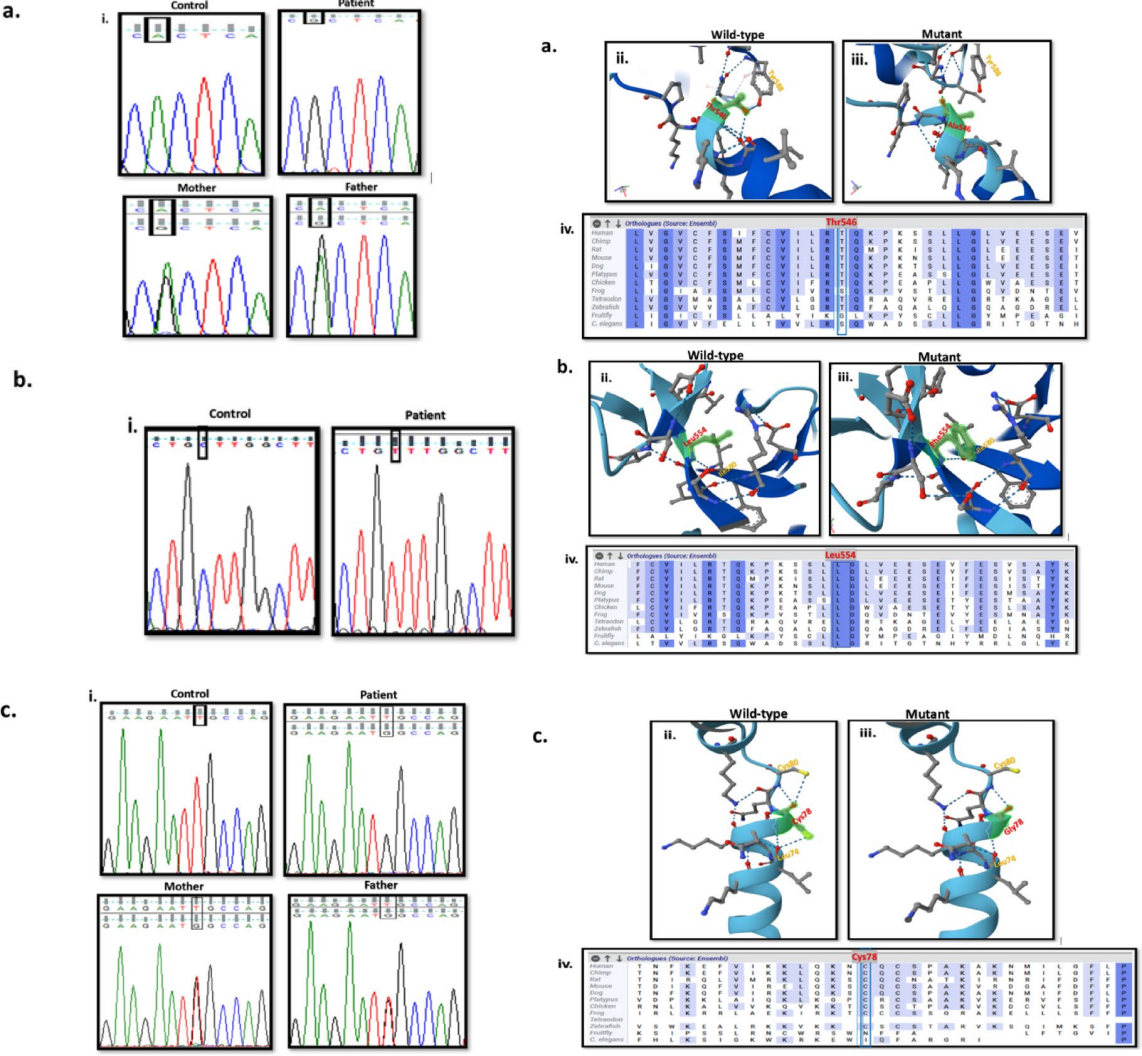

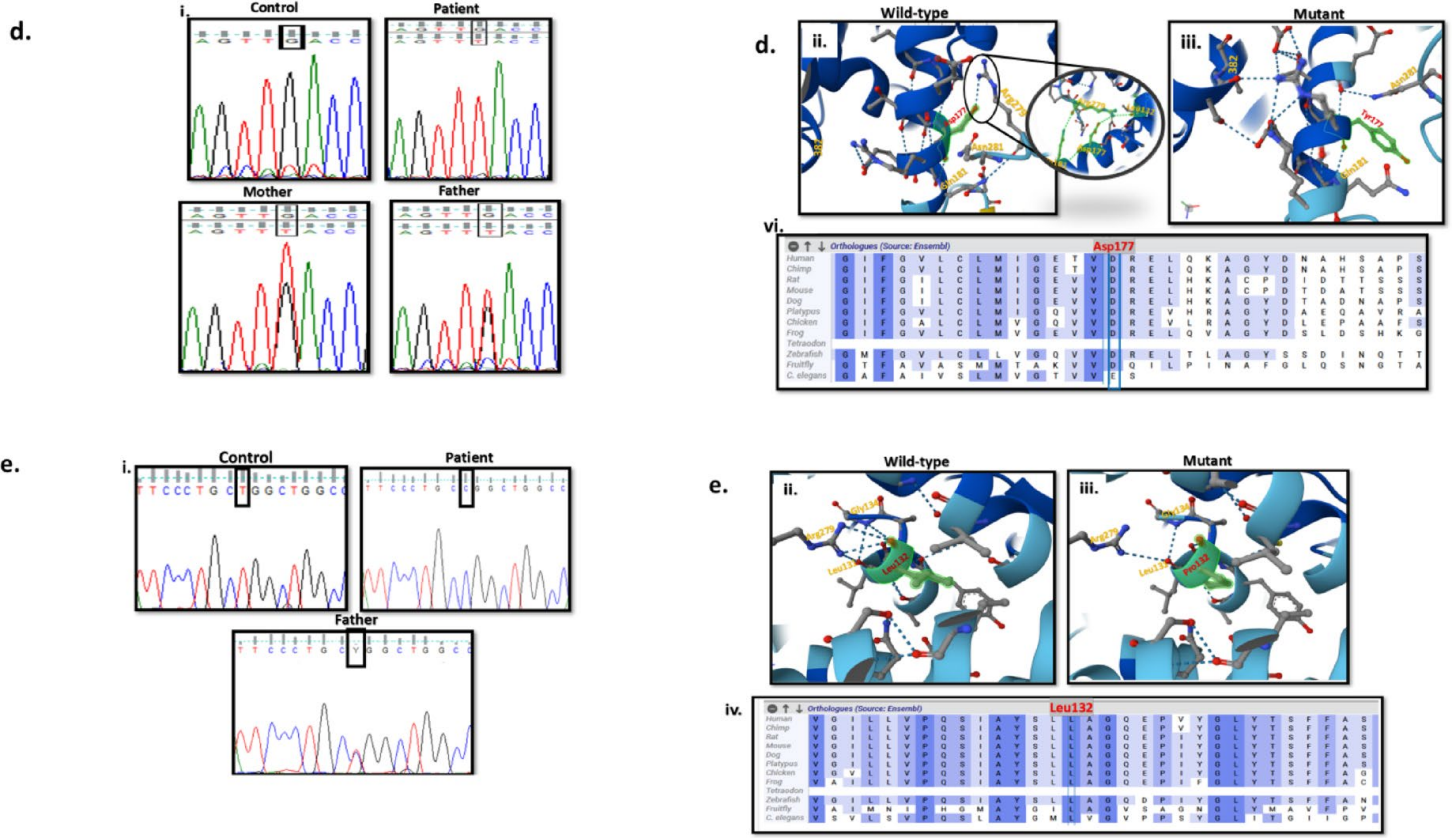
Sequencing analysis and AlphaFold 3 structural protein modelling for the five novel variants. (**a**) c.1636 A > G (p.Thr546Ala) variant in P3, (i) sequence chromatograms showing variant in control, patient, and parents, (ii) Structural model showing the wild-type Threonine forming bonding with the side chain of neighboring Tyrosine588, (iii) Structural model showing the mutant Alanine with loss of bonding, (iv) Multiple sequence alignment at the site of variation shows that Thr546 is a moderately conserved amino acid. (**b**) c.1660 C > T (p.Leu554Phe) variant in P4, (i) sequence chromatograms showing variant in control and patient, (ii) Structural model showing the wild-type Leucine, (iii) Structural model showing the mutant Phenylalanine showing the difference in the size of the side chain, (iv) Multiple sequence alignment at the site of variation shows that Leu554 is a highly conserved amino acid. (**c**) NM_000112.4:c.232T > G (p.Cys78Gly) variant in P6, (i) sequence chromatograms showing variant in control, patient, and parents, (ii) Structural model showing the wild-type Cysteine forming bonding with the side chain of neighboring residues, (iii) Structural model showing the mutant Glycine with loss of bonding, (iv) Multiple sequence alignment at the site of variation shows that Cys78 is a moderately conserved amino acid. (**d**) NM_000112.4:c.529G > T (p.Asp177Tyr) variant in P8, (i) sequence chromatograms showing variant in control, patient, and parents, (ii) Structural model showing the wild-type Aspartic acid forming bonding with the side chain of Arginine279, (iii) Structural model showing the mutant Tyrosine with forming bonding with 381, 382, and 385 residues, (iv) Multiple sequence alignment at the site of variation shows that Asp177 is a highly conserved amino acid. (**e**) NM_000112.4:c.395T > C (p.Leu132Pro) variant in P10, (i) sequence chromatograms showing variant in control, patient, and the father, (ii) Structural model showing the wild-type Leucine, (iii) Structural model showing the mutant Proline affecting the bending of the TM loop changing the proximities of residues and forming additional bonding, (iv) Multiple sequence alignment at the site of variation shows that Leu132 is a highly conserved amino acid.

In silico prediction tools were used to utilize the pathogenicity and the potential damaging effect of the novel variants^18,19,22^. Aggregated prediction for these variants was calculated and determined within the deleterious score (Table 2). Sanger sequencing of the available parents validated the novel variants (Fig. 3). Protein structures predictions for novel variants were generated by AlpaFold 3, along with protein multiple sequence alignment to identify conservation of the questioning amino acids^23^.

**Table 2:** Classification and prediction tools for patients with detected homozygous variants of the *SLC26A2* Gene.

	Variant and Protein location		ACMG classification	dbSNP	Allele frequency (gnomAD)	Aggregated Prediction**	Revel	AlphaMissense	Varity	SIFT	Polyphen2	MetaLR	BayesDel	phyloP100	CADD
P1	NM_000112.4:c.1157 C > T p.Ala386Val Exon 3	TM-8	Pathogenic [PP3, PP5, PM5,PM2, PP2]	rs386833493	ND	Deleterious (0.99)	Deleterious (Strong) (0.96)	Deleterious (Supporting) (0.854)	Deleterious (0.88)	Uncertain (0.001)	Deleterious (1)	Deleterious (0.93)	Deleterious (Strong) (0.51)	7.86	26.7
P2	NM_000112.4:c.1157 C > T p.Ala386Val Exon 3	TM-8	Pathogenic [PP3, PP5, PM5,PM2, PP2]	rs386833493	ND	Deleterious (0.99)	Deleterious (Strong) (0.96)	Deleterious (Supporting) (0.854)	Deleterious (0.88)	Uncertain (0.001)	Deleterious (1)	Deleterious (0.93)	Deleterious (Strong) (0.51)	7.86	26.7
P3	NM_000112.4:c.1636 A > G p.Thr546Ala Exon 3	TM-14	Uncertain Significance [PM2, PP3*]	rs772074266	0.00002	Deleterious (0.84)	Deleterious (Moderate) (0.79)	Benign (Supporting) (0.235)	Deleterious (0.57)	Uncertain (0.002)	Uncertain (0.8)	Deleterious (0.84)	Deleterious (Moderate) (0.41)	7.23	25.3
P4	NM_000112.4: c.1660 C > T p.Leu554Phe Exon 3	STAS	Uncertain Significance [PM2, PP3*]	NA	0.000002	Deleterious (0.7)	Deleterious (Supporting) (0.66)	Uncertain (0.456)	Deleterious (0.79)	Deleterious (Supporting) (0)	Deleterious (1)	Deleterious (0.96)	Deleterious (Supporting) (0.27)	4.06	24.9
P5	NM_000112.4: c.1120 C > T p.Pro374Ser Exon 3	EC-4	Uncertain Significance [PM2, PP3*]	rs763082940	0.00002	Deleterious (0.87)	Deleterious (Moderate) (0.9)	Deleterious (Supporting) (0.825)	Deleterious (0.89)	Deleterious (Supporting) (0)	Deleterious (1)	Deleterious (0.95)	Deleterious (Strong) (0.53)	7.86	25
P6	NM_000112.4:c.232T > G p.Cys78Gly Exon 2	N-terminal cytosolic	Uncertain Significance [PM2, PP3*]	NA	ND	Deleterious (0.87)	Deleterious (Moderate) (0.91)	Benign (Supporting) (0.163)	Deleterious (0.88)	Uncertain (0.003)	Deleterious (1)	Deleterious (0.86)	Deleterious (Moderate) (0.44)	6.24	27.1
P7	NM_000112.4:c.835 C > T p.Arg279Trp Exon 3	EC-3	Likely pathogenic [PM3, PS3, PM1, PM2, PP3, PP5]	rs104893915	0.0009	Deleterious (0.88)	Deleterious (Moderate) (0.92)	Uncertain (0.378)	Deleterious (0.96)	Deleterious (Supporting) (0)	Deleterious (1)	Deleterious (0.92)	Deleterious (Moderate) (0.47)	3.22	27
P8	NM_000112.4:c.529G > T p.Asp177Tyr Exon 2	EC-2	Uncertain Significance [PM2, PP3*]	NA	ND	Deleterious (0.84)	Deleterious (Moderate) (0.79)	Benign (Supporting) (0.223)	Deleterious (0.82)	Uncertain (0.015)	Uncertain (0.7)	Deleterious (0.88)	Deleterious (Moderate) (0.3)	9.597	27.3
P9	NM_000112.4:c.1020_1022delTGT p.Val341del Exon 3	TM-7	Pathogenic [PM3, PM4, PP5, PM2, PP5]	rs121908077	0.0001	-	-	-	-	-	-	-	-	9.94	18
P10	NM_000112.4:c.395T > C p.Leu132Pro Exon 2	TM-1	Uncertain Significance [PM2, PP3*]	rs1755026324	0.000002	Deleterious (0.9)	Deleterious (Strong) (0.97)	Deleterious (Moderate) (0.978)	Deleterious (0.99)	Deleterious (Supporting) (0)	Deleterious (1)	Deleterious (0.94)	Deleterious (Strong) (0.53)	8.01	28.8

**NA**: not available, **ND**: not detected, **TM**: transmembrane, **EC**: extracellular, **STAS**: sulfate transporter and anti-sigma factor antagonist.

* Classification is according to the latest recommendations for PP3 and BP4 criteria by Pejaver et al., 2022.

** Aggregated Prediction relies on the ensemble model combining REVEL and MetaLR.

## Discussion

Skeletal dysplasias (SDs), also known as osteochondrodysplasias, are a rare group of heterogeneous disorders characterized by cartilage and bone growth abnormalities affecting bone length, shape or density^24^. SDs are distinguished into three groups: osteodysplasias, chondrodysplasias, and dysostosis. Osteodysplasias are characterized by irregularities in bone structure, while chondrodysplasias are associated with abnormalities in cartilage. Dysostosis refers to anomalies that affect either individual bones or groups of bones, thereby impacting specific skeletal elements^3^. Differentiating among these categories is complex due to overlapping characteristics, which may manifest as shortened, bowed, or fractured long bones, hypo-ossification of long bones, calvarium, and vertebrae, as well as dysmorphic scapula, ribs, calvarium, long bones, and vertebrae. Additionally, limb deformities such as clubfoot (equinovarus), polydactyly, clinodactyly, and the absence or underdevelopment of certain bony structures, along with a reduced thoracic cavity, further complicate classification^25^. Up to date, more than 500 genes are associated with SDs^1^. Exome sequencing (ES) is recognized as a precise and effective method for identifying the genes and mutations involved in these complex conditions^26^.

Diastrophic dysplasia (DTD) is clinically characterized by limb shortening, reduced stature, and abnormal development of joints and skeletal structures in various anatomical regions. Patients with DTD may also experience progressive spinal deformities, particularly scoliosis and/or kyphosis, as well as pathological changes in the tissue of the pinnae (external ear). Additional features may include craniofacial anomalies such as cleft palate, bilateral hip dysplasia, patellar dislocation, foot deformities, and ulnar deviation of the second digit, often accompanied by thumb abduction. Respiratory complications associated with DTD can lead to elevated mortality rates among affected infants during the neonatal period^27^.

The etiology of diastrophic dysplasia (DTD) is associated with variants in the SLC26A2/DTDST gene (OMIM #606718)^28^. Variants in the SLC26A2 gene are linked to several chondrodysplasias, including autosomal recessive achondrogenesis type IB (ACG-1B), atelosteogenesis type 2 (AO2), diastrophic dysplasia (DTD), and multiple epiphyseal dysplasia type 4 (EDM-4). The presence of biallelic pathogenic variants disrupts sulfate transport across cellular membranes, resulting in inadequately sulfated proteoglycans, which subsequently leads to cartilage immaturity and skeletal deformities^12^. Although SLC26A2 protein expression has been detected in various cell types beyond chondrocytes, the phenotypic manifestations of DTD are primarily confined to chondrocyte-dependent tissues^29^.

In the present study, ES analysis of the affected patients revealed nine homozygous variants in *SLC26A2* gene in ten patients (73%), five variants were novel and four were previously reported.

Patients with novel homozygous variants are patients (P3, P4, P6, P8, and P10). They were three females and two males, who are offspring of consanguineous parents. The five patients presented with short stature, with a height standard deviation score (SDS) of -6.2 ± 1.9. They also have short long bones, rhizomelia, hitchhiker thumbs, and joint contractures. All patients exhibited well-characterized clinical and skeletal deformities associated with DTD, consistent with previously reported disease manifestations^13^.

The remaining five unrelated patients (P1, P2, P5, P7, and P9) carried four homozygous previously reported variants in the *SLC26A2* gene. Patient 7 (P7) is observed with cleft palate, hitchhiker thumbs, and cystic swelling of the outer ear which are clinically consistent with DTD. While previous reports of patients with homozygous or compound heterozygous p.Arg279Trp variants described only early MED-4 manifestations^30^, our findings expand the phenotypic spectrum of the homozygous p.Arg279Trp variant. The reported cases were characterized by short stature (-1.24 SD to -4.08 SD), progressive contractures in the large joints, and limb shortening. While, brachydactyly, and congenital bilateral clubfoot were less common^8,11,30^. Additionally, compound heterozygous genotype with mutations such as the Finnish founder variants (c.-26 + 2T > C), p.Arg178X, or p.Gly663Arg, p.Arg279Trp is linked to classical diastrophic dysplasia (DTD) or intermediate forms between DTD and atelosteogenesis type 2 (AO2)^7,31^.

The homozygous deletion variant c.1020_1022delTGT was first reported to cause ACG-IB^32^. Compound heterozygous c.1020_1022del and c.1262T > C variants in the *SLC26A2* gene were identified to be the cause of MED-4^33^. It was also reported in a 42 years old woman with diastrophic dysplasia (DTD)^10^. There is wide spectrum of manifestations for the same variant. However, the phenotype of patients with homozygous deletion variants is more severe than that of patients with compound heterozygous variants with missense ones, which is consistent with the phenotype of P9.

In patient P5, a homozygous p.Pro374Ser variant was detected which was previously reported once in a study of a cohort of patients with skeletal dysplasias, homozygous p.Pro374Ser was identified in a patient with suspected diastrophic dysplasia^34^. The site of this variant is within the extracellular domain 3 (EC-3) in highly conserved site, and of high pathogenicity prediction score. Although reported by *Scocchia et al.*., p.Pro374Ser remains classified as a variant of uncertain significance (VUS) due to insufficient functional validation.

The three previously reported variants were in exon 3 within the transmembrane and extracellular domains. The p.Ala386Val variant in P1 and P2 and the c.1020_1022delTGT in P9 were within the transmembrane 8 and 7 helices (TM-8, TM-7), respectively. Whereas the p.Pro374Ser in P5 and the p.Arg279Trp in P7 are located in extracellular 4 and 3 loops (EC-4, EC-3), respectively.

The p.Arg279Trp variant is considered to be the most common pathogenic variant in Caucasians and is the second most frequent variant in Finnish people^27^. Functional studies identified that the p.Arg279Trp and the p.Ala386Val variants reduced the transported sulfate and oxalate at rates of 20 to 30% of wild-type protein. The surface abundance of the protein was reduced by the p.Ala386Val variant, but the surface abundance was at wild-type levels in p.Arg279Trp mutant protein^28,29^.

The DTDST protein is characterized by 14 transmembrane helices (TM1-14), seven extracellular loops (EC1-7), and six cytosolic loops, culminating in a cytoplasmic sulfate transporter and anti-sigma factor antagonist (STAS) domain at the C-terminus^30^. Pathogenic variants predominantly disrupt sulfate uptake, with most mutations localized within the transmembrane domains and the STAS domain^11^.

Novel missense variants were in exons 2 and 3 (p.Cys78Gly, p.Leu132Pro, p.Asp177Tyr, p.Thr546Ala, p.Leu554Phe). Only the second variant (p.Leu132Pro) was previously submitted to ClinVar (accession number: RCV003237558). The pathogenicity of identified variants was of strong supporting evidence with scores ranges from (0.7 to 0.9). The alternative allele frequencies were extremely low (below 0.00002), without detecting any homozygous alleles for any of the five variants in the population databases (Table 2). The novel variations showed moderately to highly conserved nucleotides with PhyloP100 ranges from 4.1 to 9.6, and moderately to highly conserved amino acids. On the other hand, sequencing analysis of the available parents showed heterozygous variations patterns (Fig. 3).

The size of the mutant amino acids was smaller than the wild-type in p.Cys78Gly, p.Leu132Pro, and p.Thr546Ala variants. These three variants are located in the N-terminal cytosolic domain, transmembrane helix 1 (TM-1), and transmembrane helix 14 (TM-14). Structural modeling revealed that these variants disrupt hydrogen bonding with neighboring residues, which may induce conformational changes and impair external protein interactions. Notably, the α-helices are stabilized by backbone hydrogen bonds. While the other two variants p.Asp177Tyr, and p.Leu554Phe, the mutant residues are bigger than the wild-type ones, converting aliphatic side chain with aromatic one. In p.Asp177Tyr, this change disrupts hydrogen bonding with Arg279 while forming new interactions with residues 381, 382, and 385, therefore, this alteration potentially destabilizes the extracellular loop 2 (EC-2) interactions and function. In contrast, the p.Leu554Phe variant substitutes one hydrophobic residue for another (Leu→Phe) at a conserved position within a β-sheet of the STAS domain core. While this substitution is unlikely to induce major conformational changes, molecular modeling predicts that the bulkier phenylalanine side chain may disrupt the optimal Van der Waals faces that packing in the hydrophobic core, and consequently destabilize STAS domain architecture^35^.

While the current study focuses on the in silico structural characterization of the novel variants, their functional validation should be addressed in subsequent investigations. Experimental analyses of these variants will be essential to confirm their biochemical and physiological impacts.

No detected variants related to the *SLC26A2* gene have identified in five cases (P11-15) despite the overlapping clinical findings (Supp. Table 1). Exome sequencing did not detect any pathogenic, likely pathogenic, or uncertain significant variants related to their phenotypes. Differentiation between the groups of chondrodysplasias is complex because of the overlapping features^36^. Although there are known types such as Desbuquois dysplasia-1 (MIM# 251450), Larsen syndrome (MIM# 150250), Spondyloepiphyseal dysplasia with congenital joint dislocations (MIM#143095), which could show pseudodiastrophic dysplasia phenotype (MIM#264180)^37^, the molecular diagnosis could not be determined for those five patients.

In conclusion, this study is the first to report the clinical and molecular findings in Egyptian patients with DTD. The report of novel variants in the *SLC26A2* gene expands the mutational spectrum of DTD. Although in silico and molecular modeling approaches can predict novel variant pathogenicity, experimental studies are crucial to elucidate disease mechanisms. Additional functional studies are therefore recommended to verify computational predictions and further explore the biological consequences. The molecular diagnosis of five patients has not been established, despite the exome sequencing’s capacity to detect the causative variants. This may be attributed to novel genes that require further research to confirm or may be because of the location of the causative variants that are outside the scope of the ES, such as copy number variations, deep intronic variants, or exonic deletions.

## Supplementary Information

Below is the link to the electronic supplementary material.

Supplementary Material 1

## Data Availability

The datasets generated and/or analyzed during the current study are available in ClinVar with Accession numbers SCV005911665, SCV005911664, SCV005911663, SCV005911662, SCV005911661, SCV005911660, SCV005911659, SCV005911658, SCV005911657.

## References

[1] Unger, S. et al. Nosology of genetic skeletal disorders: 2023 revision. *Am. J. Med. Genet. A*. **191** (5), 1164–1209 (2023). 36779427 10.1002/ajmg.a.63132 PMC10081954

[2] Colares Neto, G. P. & Alves, C. A. D. *Desmistifying Skeletal Dysplasias: A Practical Approach for the Pediatric Endocrinologist* (Horm Res Paediatr, 2024). 10.1159/000536564 38310868

[3] Hsu, R. H. et al. Next-generation sequencing identifies TRPV4-related skeletal dysplasia in a Boy with progressive Bowlegs. *Pediatr. Neonatol*. **60** (1), 102–104 (2019). 29776788 10.1016/j.pedneo.2018.04.002

[4] Victoria, T. et al. What is new in prenatal skeletal dysplasias? *AJR Am. J. Roentgenol.* **210** (5), 1022–1033 (2018). 29528710 10.2214/AJR.17.19337

[5] Agirdil, Y. The growth plate: a physiologic overview. *EFORT Open. Rev.* **5** (8), 498–507 (2020). 32953135 10.1302/2058-5241.5.190088 PMC7484711

[6] Handa, A. et al. Skeletal dysplasia families: A Stepwise approach to diagnosis. *Radiographics* **43** (5), e220067 (2023). 37053103 10.1148/rg.220067

[7] Harkonen, H. et al. SLC26A2-Associated Diastrophic Dysplasia and rMED-Clinical Features in Affected Finnish Children and Review of the Literature. Genes (Basel) 12 (5). (2021). 10.3390/genes12050714 PMC8151170 34064542

[8] Barreda-Bonis, A. C. et al. Multiple SLC26A2 mutations occurring in a three-generational family. *Eur. J. Med. Genet.* **61** (1), 24–28 (2018). 29024831 10.1016/j.ejmg.2017.10.007

[9] Zheng, C. et al. Suppressing UPR-dependent overactivation of FGFR3 signaling ameliorates SLC26A2-deficient chondrodysplasias. *EBioMedicine* **40**, 695–709 (2019). 30685387 10.1016/j.ebiom.2019.01.010 PMC6413327

[10] Bondarenko, M. et al. SLC26A2 related diastrophic dysplasia in 42-Years Ukrainian women. *Balkan J. Med. Genet.* **25** (2), 83–90 (2023). 37265969 10.2478/bjmg-2022-0018 PMC10230836

[11] Silveira, C. et al. SLC26A2/DTDST spectrum: A cohort of 12 patients associated with a comprehensive review of the Genotype-Phenotype correlation. *Mol. Syndromol*. **13** (6), 485–495 (2023). 36660027 10.1159/000525020 PMC9843583

[12] Kimball, T. N. et al. Esophageal stenosis in an adult Mexican patient with diastrophic dysplasia: case report. *Clin. Case Rep.* **11** (10), e8028. (2023). 10.1002/ccr3.8028 PMC10593974 37881199

[13] Unger, S. & Superti-Furga, A. Diastrophic dysplasia. (2021). 20301524

[14] Haghighi, A. et al. An integrated clinical program and crowdsourcing strategy for genomic sequencing and Mendelian disease gene discovery. *NPJ Genom Med.* **3**, 21 (2018). 30131872 10.1038/s41525-018-0060-9 PMC6089983

[15] Marshall, C. R. et al. Best practices for the analytical validation of clinical whole-genome sequencing intended for the diagnosis of germline disease. *NPJ Genom Med.* **5**, 47 (2020). 33110627 10.1038/s41525-020-00154-9 PMC7585436

[16] Pounraja, V. K. et al. A machine-learning approach for accurate detection of copy number variants from exome sequencing. *Genome Res.* **29** (7), 1134–1143 (2019). 31171634 10.1101/gr.245928.118 PMC6633262

[17] Bhaskaran, S. & Saikumar, C. A review of next generation sequencing methods and its applications in laboratory diagnosis. *J. Pure Appl. Microbiol.* **16** (2). (2022).

[18] Dong, C. et al. Comparison and integration of deleteriousness prediction methods for nonsynonymous SNVs in whole exome sequencing studies. *Hum. Mol. Genet.* **24** (8), 2125–2137 (2015). 25552646 10.1093/hmg/ddu733 PMC4375422

[19] Ioannidis, N. M. et al. REVEL: an ensemble method for predicting the pathogenicity of rare missense variants. *Am. J. Hum. Genet.* **99** (4), 877–885 (2016). 27666373 10.1016/j.ajhg.2016.08.016 PMC5065685

[20] Richards, S. et al. Standards and guidelines for the interpretation of sequence variants: a joint consensus recommendation of the American college of medical genetics and genomics and the association for molecular pathology. *Genet. Med.* **17** (5), 405–424 (2015). 25741868 10.1038/gim.2015.30 PMC4544753

[21] Pejaver, V. et al. Calibration of computational tools for missense variant pathogenicity classification and ClinGen recommendations for PP3/BP4 criteria. *Am. J. Hum. Genet.* **109** (12), 2163–2177 (2022). 36413997 10.1016/j.ajhg.2022.10.013 PMC9748256

[22] Rentzsch, P. et al. CADD: predicting the deleteriousness of variants throughout the human genome. *Nucleic Acids Res.* **47** (D1), D886–D894 (2019). 30371827 10.1093/nar/gky1016 PMC6323892

[23] Abramson, J. et al. Accurate structure prediction of biomolecular interactions with alphafold 3. *Nature* **630** (8016), 493–500 (2024). 38718835 10.1038/s41586-024-07487-w PMC11168924

[24] Marzin, P. & Cormier-Daire, V. New perspectives on the treatment of skeletal dysplasia. *Ther. Adv. Endocrinol. Metab.* **11**, 2042018820904016 (2020). 32166011 10.1177/2042018820904016 PMC7054735

[25] Chang, T. Y. et al. Whole exome sequencing with comprehensive gene set analysis identified a biparental-origin homozygous c. 509G > A mutation in PPIB gene clustered in two Taiwanese families exhibiting fetal skeletal dysplasia during prenatal ultrasound. *Diagnostics* **10** (5), 286 (2020). 32392875 10.3390/diagnostics10050286 PMC7277976

[26] Chandler, N. et al. Rapid prenatal diagnosis using targeted exome sequencing: a cohort study to assess feasibility and potential impact on prenatal counseling and pregnancy management. *Genet. Med.* **20** (11), 1430–1437 (2018). 29595812 10.1038/gim.2018.30

[27] Rossi, A. & Superti-Furga, A. Mutations in the diastrophic dysplasia sulfate transporter (DTDST) gene (SLC26A2): 22 novel mutations, mutation review, associated skeletal phenotypes, and diagnostic relevance. *Hum. Mutat.* **17** (3), 159–171 (2001). 11241838 10.1002/humu.1

[28] Heneghan, J. F. et al. Regulated transport of sulfate and oxalate by SLC26A2/DTDST. *Am. J. Physiol. Cell. Physiol.* **298** (6), C1363–C1375 (2010). 20219950 10.1152/ajpcell.00004.2010 PMC2889644

[29] Karniski, L. P. Functional expression and cellular distribution of diastrophic dysplasia sulfate transporter (DTDST) gene mutations in HEK cells. *Hum. Mol. Genet.* **13** (19), 2165–2171 (2004). 15294877 10.1093/hmg/ddh242

[30] Markova, T. et al. Clinical and Genetic Characteristics of Multiple Epiphyseal Dysplasia Type 4. Genes (Basel) 13 (9). (2022). 10.3390/genes13091512 PMC9498659 36140680

[31] Barbosa, M. et al. Clinical and molecular characterization of diastrophic dysplasia in the Portuguese population. *Clin. Genet.* **80** (6), 550–557 (2011). 21155763 10.1111/j.1399-0004.2010.01595.x

[32] Superti-Furga, A. et al. Achondrogenesis type IB is caused by mutations in the diastrophic dysplasia sulphate transporter gene. *Nat. Genet.* **12** (1), 100–102 (1996). 8528239 10.1038/ng0196-100

[33] Li, S. et al. Biallelic variants in SLC26A2 cause multiple epiphyseal dysplasia-4 by disturbing chondrocyte homeostasis. *Orphanet J. Rare Dis.* **19** (1), 245 (2024). 38956600 10.1186/s13023-024-03228-4 PMC11220988

[34] Scocchia, A. et al. Diagnostic utility of next-generation sequencing-based panel testing in 543 patients with suspected skeletal dysplasia. *Orphanet J. Rare Dis.* **16** (1), 412 (2021). 34627339 10.1186/s13023-021-02025-7 PMC8501536

[35] Rapp, C. et al. Molecular analysis of human solute carrier SLC26 anion transporter disease-causing mutations using 3-dimensional homology modeling. *Biochim. Biophys. Acta Biomembr.* **1859** (12), 2420–2434 (2017). 28941661 10.1016/j.bbamem.2017.09.016

[36] Cormier, A. A. et al. Overlapping genetic pathways in the skeletal dysplasias of a middle woodland individual: A case study. *Int. J. Paleopathol.* **18**, 98–107 (2017). 28888399 10.1016/j.ijpp.2017.06.001

[37] Piwar, H. et al. Clinical and genetic insights into desbuquois dysplasia: review of 111 case reports. *Int. J. Mol. Sci.* **25**, 17 (2024). 10.3390/ijms25179700 PMC11395126 39273648

