# Supplementary material for: Clinical and molecular characterization of chondrodysplasias in a cohort of Egyptian patients
Source: Sci Rep. 2025 Oct 29;15:37751. doi: 10.1038/s41598-025-22794-6 (PMC12572108; doi:10.1038/s41598-025-22794-6)
Supplement: Supplementary file 1 — Supplementary Material 1 [file 41598_2025_22794_MOESM1_ESM.docx]

| **Criteria** | **P11** | **P12** | **P13** | **P14** | **P15** |
| --- | --- | --- | --- | --- | --- |
| **Age at examination** | 4 months | 2 months | 17 years | 2 months | 4 years |
| **Sex** | M | M | M | M | M |
| **Parental consanguinity** | +ve | +ve | +ve | -ve | +ve |
| **Similarly affected family members** | -ve | -ve | -ve | -ve | -ve |
| **Motor milestones** | Not yet | Not yet | Delayed | Not yet | Delayed |
| **Height SDS** | -3.2 | -2.0 | -4.2 | -5.85 | -3.7 |
| **Disproportionate short stature** | Short limbs dwarfism | Short limbs dwarfism | Short limbs dwarfism | Short limbs dwarfism | Short limbs dwarfism |
| **Segmental shortening of long bones** | normal | acromelia | mesomelia | rhizomelia | rhizomelia |
| **Glabellar hemangioma** | **Facial features** |  |  |  |  |
| **Ear pinnae** | -ve | -ve | -ve | -ve | -ve |
| **Cleft palate** | Thick, cauliflower, ossified mass | normal | Right ear: cauliflower shape due to ear cartilage enlargement | Thick ear pinnae | Normal |
| **Adducted (Hitchhiker) thumbs** | -ve | -ve | -ve | -ve | -ve |
| **Brachydactyly** | **Skeletal deformities (clinical & radiological)** |  |  |  |  |
| **Nails** | +ve | +ve | -ve  Dislocated 1^st^ metacarpals | +ve | -ve |
| **Talipes** | -ve | +ve | +ve | -ve | -ve |
| **Broad 1^st^ toes** | normal | dysplastic | normal | normal | normal |
| **Joint dislocations** | +ve | -ve | +ve | +ve | -ve |
| **Contracture deformities** | -ve | +ve | +ve, long | +ve | -ve |
| **Short broad Long bones (radiological)** | Hip | Elbows, interphalangeal joints | Hips | -ve | -ve |
| **Epiphysis (radiological)** | +ve | Elbows and knees | Elbows, hip and knees | -ve | -ve |
| **Metaphysis (radiological** | +ve | +ve | +ve | +ve | +ve |
| **Spine** | Delayed ossification | Flat, dysplastic, fragmented, absent head of femurs | Deformed | Delayed ossification | Deformed |
| **Vertebrae**  **(radiological)** | Broad | Broad | Mildly broad | Frayed, cupped | Broad |
| **Flat acetabulum (radiological)** | Scoliosis | Kyphosis | normal | Kypho-Scoliosis | normal |
| **Others** | Irregular vertebral end plate | Irregular end plate, platyspondyly with anterior beaking, and spina bifida | Platyspondyly,  Irregul,ar vertebral end plate | Platyspondyly with anterior beaking | Irregular vertebral end plate |
|  | +ve | +ve | +ve | +ve | -ve |
|  | Pectus excavatum | Pectus carinatum | Bowed femurs | - | - |

**Supp. Table 1: Clinical characteristics of the studied cases with negative genetic results**
